# Investigating SMYD3 role during oocyte maturation in a 3D follicle-enclosed oocyte *in vitro* model in sheep

**DOI:** 10.3389/fcell.2025.1625914

**Published:** 2025-06-25

**Authors:** Chiara Camerano Spelta Rapini, Alessia Peserico, Chiara Di Berardino, Giulia Capacchietti, Camila Rojo-Fleming, Andrada-Ioana Damian-Buda, Irem Unalan, Aldo Roberto Boccaccini, Valentina Grossi, Mauro Mattioli, Barbara Barboni

**Affiliations:** ^1^ Department of Bioscience and Technology for Food, Agriculture and Environment, University of Teramo, Teramo, Italy; ^2^ Department of Materials Science and Engineering, Institute of Biomaterials, University of Erlangen-Nuremberg, Erlangen, Germany; ^3^ Medical Genetics, National Institute of Gastroenterology, IRCCS “Saverio de Bellis” Research Hospital, Castellana Grotte (Ba), Italy

**Keywords:** SMYD3, follicle-enclosed oocyte *in vitro* maturation (FEO-IVM), ovine oocyte competence, cumulus-oocyte metabolic coupling, hCG-dependent signaling pathways

## Abstract

**Background:**

SMYD3 is a histone methyltransferase known for its dual role in modifying both histone and non-histone proteins. Despite its established involvement in somatic cell function and oncogenesis, its role in mammalian oogenesis and early embryonic development remains unclear. This study aimed to elucidate the function of SMYD3 in regulating oocyte meiotic progression and developmental competence using sheep as a mono-ovulatory model.

**Results:**

Utilizing a 3D follicle-enclosed *in vitro* maturation (FEO-IVM) system, the study examined the impact of SMYD3 inhibition on oocyte maturation within Early Antral follicles In the absence of human chorionic gonadotropin oocytes remained arrested at the germinal vesicle (GV) stage. Interestingly, treatment with a SMYD3 inhibitor (iSMYD3) alone prompted germinal vesicle breakdown (GVBD) in 67% of oocytes; however, progression to the metaphase II (MII) stage was achieved only when iSMYD3 was combined with hCG, resulting in a 73% maturation rate. Despite this, MII oocytes from the iSMYD3 group exhibited compromised developmental competence, as evidenced by the failure of parthenogenetic embryos to progress beyond the 8-cell stage, contrasting with a 29% success rate in the hCG-only group. At the molecular level, SMYD3 inhibition led to sustained activation of CDC25A within oocytes, facilitating GVBD but impeding the MI-MII transition due to the absence of CDC25A degradation. Moreover, iSMYD3 failed to activate the MAPK1/3 and PDE5A pathways in the somatic compartment, unlike hCG treatment, indicating distinct signaling mechanisms. Additionally, hCG rapidly downregulated SMYD3 expression in follicular walls and cumulus cells, a process independent of meiotic progression but essential for metabolic decoupling between oocytes and cumulus cells. SMYD3 inhibition disrupted this decoupling by preventing hCG-induced gap junction closure, thereby maintaining prolonged intercellular communication.

**Conclusion:**

SMYD3 is identified as a key modulator of oocyte maturation, orchestrating meiotic progression through CDC25A regulation and interacting with hCG-driven somatic signaling. These findings highlight SMYD3 as a critical determinant of late oogenesis and a potential target for enhancing oocyte competence in assisted reproductive technologies

## 1 Introduction

A mature oocyte possesses all the molecular machinery to express a complete developmental competence leading to totipotency after fertilization (Conti and Franciosi, 2018; Clift and Schuh, 2013). The understanding of the mechanisms involved in this remarkable biological milestone, achieved through a highly orchestrated process, remains a challenge for advancing knowledge of reproductive biology and for having a positive impact on the related assisted reproductive technologies (ART).

Physiologically, the acquisition of developmental competence occurs during the final phase of oocyte maturation, triggered by the preovulatory surge of gonadotropins. This hormonal cue initiates a cascade of coordinated nuclear and cytoplasmic events that equip the oocyte to undergo successful fertilization and support early embryonic cleavage (Conti and Franciosi, 2018). While this process is known to involve extensive molecular remodeling, including epigenetic reprogramming (Solc et al., 2008; Lincoln et al., 2002; Ferencova et al., 2022), the specific molecular drivers of meiotic progression and their temporal regulation remain incompletely understood.

Among the regulatory proteins expressed during oogenesis, SMYD3 has recently emerged as a molecule of interest (Suzuki et al., 2015; Bai et al., 2016). A member of the SMYD (SET and MYND domain-containing) protein family, SMYD3 has been broadly studied in somatic contexts for its role in transcriptional regulation and intracellular signaling (Hamamoto et al., 2004). However, its functional role within the oocyte while still enclosed in the somatic follicular environment, especially during meiotic resumption and maturation, remains to be fully elucidated. Given its dynamic expression profile in the ovary and potential responsiveness to hormonal cues, SMYD3 may represent a novel factor involved in the orchestration of late-stage oocyte maturation.

In both mouse and bovine embryos, SMYD3 mRNA expression rises post-fertilization, peaking during the initial cell divisions in mice (Suzuki et al., 2015) and at the morula stage in bovines (Bai et al., 2016), followed by a reduction at the blastocyst stage in both species. Knockdown studies in mice have further revealed SMYD3’s critical involvement in peri-implantation development, influencing key regulatory genes such as *Oct4* and *Cdx2* (Suzuki et al., 2015). Conversely, knowledge about SMYD3’s role in oogenesis is limited. Studies have primarily focused on its transcript-level expression in oocytes across various species, including mice, bovines, humans, and felines, suggesting a conserved role in gamete development (Suzuki et al., 2015; Bai et al., 2016; Oliveri et al., 2007; Phillips et al., 2016). More specifically, SMYD3-mediated histone methylation appears to contribute to epigenetic reprogramming during oocyte growth, as observed in both mouse and cat models (Oliveri et al., 2007; Phillips et al., 2016). Despite these insights, its precise functional contributions to oocyte maturation, the process that provides the oocyte with full developmental competence, remain unclear.

A critical challenge in studying the role of SMYD3 in oocyte maturation *in vitro* arises from the difficulty of reproducing the physiological mechanisms once the oocytes are removed from their follicular environment, as meiotic division resumes spontaneously and independently of regulatory control. Consequently, although nuclear maturation proceeds, the absence of specific cues to trigger cytoplasmic and epigenetic processes may result in incomplete or aberrant maturation. This discrepancy underscores the importance of the somatic compartment in coordinating maturation, beginning with gonadotropin sensing and continuing until ovulation by synchronizing intracellular processes essential for developmental competence.

At the cellular level, junctional metabolic coupling between the somatic and germinal compartments plays a key role in ensuring the oocyte’s progression toward developmental competence, facilitating a dynamic and reciprocal exchange of signals throughout the maturation phase. Unfortunately, conventional *in vitro* models often fail to preserve this critical interaction, limiting their ability to faithfully replicate the physiological process and support the generation of high-quality matured oocytes. In the present research, we aimed to elucidate the role of SMYD3 during the critical phase of oocyte maturation by employing the Follicle-Enclosed Oocyte (FEO) *in vitro* maturation (IVM) system, using hCG as the hormonal stimulus. Due to its luteinizing hormone (LH)-like activity and residual follicle-stimulating hormone (FSH)-like effects, hCG has proven particularly effective in supporting oocyte maturation within the ovine FEO-IVM model, as demonstrated in our previous studies (Peserico et al., 2023; Di Berardino et al., 2024). This system closely recapitulates the gonadotropin-mediated signaling cascade, allowing the intrinsic meiotic inhibition of the follicular environment to be overcome while preserving the essential bidirectional communication between somatic and germinal compartments.

Early Antral follicles (EAfs) were selected as the model system because they are the earliest gonadotropin-responsive follicles amenable to whole-follicle culture under defined conditions. They have already initiated key developmental processes—such as chromatin remodeling, telomere dynamics, and cytoplasmic maturation (Colosimo et al., 2009; Barboni et al., 2011; Russo et al., 2007; Russo et al., 2006)—despite these processes remain incomplete, resulting in a reduced developmental competence compared to oocytes from later antral stages. However, this intermediate maturation state offers a unique opportunity to investigate the molecular mechanisms regulating the transition from meiotic arrest to full oocyte maturation.

Moreover, EAfs are particularly relevant in the context of advanced *in vitro* folliculogenesis, as they represent the final follicular stage capable of reliably yielding an expandable pool of oocytes suitable for IVM (Peserico et al., 2023; Di Berardino et al., 2024). Their use in this study provided a physiologically relevant and tractable model to explore the role of SMYD3 in oocyte maturation.

To investigate SMYD3’s functional involvement, we combined the FEO-IVM system with pharmacological inhibition using EPZ031686, a specific inhibitor of the SMYD3 methyltransferase (iSMYD3) (Sanese et al., 2020; Rubio-Tomás, 2021; Bottino et al., 2020). This approach enabled an in-depth analysis of the mechanisms underlying meiotic resumption and the acquisition of developmental competence in EAf-derived oocytes.

Taken together, our strategy provides a powerful model to dissect the role of SMYD3 during oocyte maturation, integrating a biologically relevant culture system with targeted molecular interrogation.

## 2 Materials and methods

### 2.1 Chemicals

Unless otherwise specified, all chemicals used in this study were purchased from Sigma-Aldrich (Sigma Chemical Co., St. Louis, MO, United States).

### 2.2 Ethical issues

No ethical concerns were involved, as the biological materials used were sourced from tissues discarded by a local slaughterhouse belonging to the food supply chain.

### 2.3 Biological samples recovery and isolation of EA follicles

Ovaries were collected from 5-month-old Appenninica sheep as per Peserico et al. (2023); Di Berardino et al. (2022). The healthy EAf were mechanically isolated and selected based on morphology and size as described by Peserico et al. (2023).

### 2.4 Follicle enclosed oocyte (FEO) IVM protocol for early antral follicles

Selected healthy EAf were cultured in trans-well co-culture systems using 96-well plates with U-shaped wells containing PCL-patterned electrospun scaffolds, as previously validated (Peserico et al., 2023; Di Berardino et al., 2022). Each system was filled with 100 µL of maturation medium composed of alpha MEM (Cat. No. BE02-002F, Lonza), 20% fetal bovine serum (Cat. No. 11573397, Gibco), 1% glutamine (Cat. No. BE17-605E/U1, Lonza), 75 mg/L penicillin-G, 50 mg/L streptomycin sulfate (Cat. No. DE17-602E, Lonza), and human Chorionic Gonadotropin (hCG, equivalent to 6 μg/mL Chorulon®, MSD Animal Health S. r.l., Segrate, Italy).

Healthy EAf were randomly assigned to one of the following FEO-IVM protocols (Conti and Franciosi, 2018): without hCG (w/o hCG) (Clift and Schuh, 2013); 25 IU/mL hCG (Solc et al., 2008); 200 µM iSMYD3 (Lincoln et al., 2002); a combination of iSMYD3 + hCG.

Cultures were maintained at 38.5°C with 5% CO_2_ for 24h. Each experimental group included at least 30 EAf samples per replicate.

To determine the optimal concentration of iSMYD3 for FEO-IVM, a preliminary dose–response curve was established using the SMYD3 inhibitor EPZ031686 (Cat. No. HY-19324, MedChemExpress LLC, Monmouth Junction, NJ, United States) at concentrations of 0, 40, 200, and 1,000 µM. The inhibitor was added during the first 18 h of FEO-IVM culture and removed for the remaining 6 h, during which hCG remained in the medium. The inhibitor was removed after 18 h to replicate the physiological downregulation of SMYD3 observed during maturation and to avoid interference with later somatic–germinal uncoupling events. The effect of the inhibitor on SMYD3 activity was assessed by evaluating the methylation status of the SMYD3 target histone mark H3K4Me2 in the somatic compartment (follicular walls and cumulus cells), as detailed in the Supplementary Figure S1.

### 2.5 Meiotic and developmental competence of FEO-IVM oocytes

#### 2.5.1 Meiotic resumption rate

Meiotic resumption in different groups was assessed by determining the nuclear stage of enclosed EAf oocytes after 24 h of incubation. Oocytes were denuded of cumulus cells, and their nuclei were counterstained with Hoechst 33342 (Cat. No. H1399 Thermo Fisher Scientific) at a 1:100 dilution in phosphate buffered saline (PBS) for 15 min. The oocytes were then analyzed under a fluorescent microscope (Jena, Germany) to classify meiotic nuclear stages into Germinal Vesicle (GV), Germinal Vesclicle BreakDown (GVBD)/Methaphase I (MI) and Metaphase II (MII) (Peserico et al., 2023; Di Berardino et al., 2022).

#### 2.5.2 Parthenogenetic activation rate

Parthenogenesis was performed and results were analyzed according to Peserico et al. (2023) to assess the cytoplasmic maturation of oocytes from the three EAf FEO-IVM groups with (1) hCG (82 MII), (Clift and Schuh, 2013), iSMYD3 (93 MII), and (Solc et al., 2008) iSMYD3 + hCG (93 MII). (Peserico et al., 2023; Di Berardino et al., 2022).

### 2.6 Biochemical detection of meiotic resumption signaling molecules

#### 2.6.1 Protein expression

Protein extracts were obtained from 30 EA-follicular walls per treatment group and homogenized in RIPA buffer with protease and phosphatase inhibitors. Total protein was extracted from 4.5 × 10^4^ cumulus cells and from 30 oocytes per treatment group in lysis buffer, supplemented with protease and phosphatase inhibitors. Protein concentration was measured with Quick Start™ Bradford Dye Reagent (Bio-Rad Laboratories, Hercules, CA, United States) and processed by SDS-PAGE and immunoblotting. Primary and secondary antibodies were used as described in Supplementary Table S1. Chemiluminescence was detected using the ChemiDoc MP Imaging System (Bio-Rad Laboratories, Hercules, CA, United States), and densitometric analysis was performed with ImageJ software (NIH, Bethesda, MD, United States).

#### 2.6.2 H3K4 methyltransferase activity assay

H3K4 methyltransferase activity was evaluated using the EpiSeeker Histone H3-K4 Methyltransferase Activity Quantification Assay Kit (Colorimetric) (Cat. No. ab113452, Abcam, Cambridge, MA, United States), following the manufacturer’s protocol. Total protein lysates were prepared from cumulus cells and follicular walls collected at specific time points (9, 12, 15 and 18 h) during FEO-IVM under hCG or iSMYD3 conditions. For each sample, 10 µg of total protein was used per well. Absorbance at 450 nm was recorded using a microplate reader (INNO-S LTEK Co. Ltd.). Data were normalized to the enzymatic activity measured at 9 h.

### 2.7 Oocyte glucose uptake detection assessed with confocal microscopy

The metabolic coupling between cumulus cells and oocytes was evaluated by tracking the transfer of fluorescent glucose (2-NBDG, Cat. No. N13195, Thermo Fisher Scientific) from the somatic to germinal compartments of cumulus-oocyte complexes (COCs) isolated from EAf after 18 h of incubation with FEO-IVM (post-GVBD).

A total of 60 COCs were incubated under two different FEO-IVM conditions: hCG and iSMYD3. For the positive control, 30 EA-COCs from FEO without hormonal stimulation (w/o hCG) were used, and denuded oocytes from 30 EA follicles served as the negative control (Ctrl -).

After incubation, COCs or oocytes were exposed to 2-NBDG (200 µM) for 30 min, then COCs were stripped of cumulus cells, and oocytes were stained with Hoechst 33,342 (Cat. No. H1399, Thermo Fisher Scientific) for nuclear labeling. The samples were visualized using a Nikon confocal microscope (Nikon Arl, Düsseldorf, Germany) equipped with NIS-Element software 4.40 (Nikon, Düsseldorf, Germany).

Three sections from the confocal z-stack (at 5 µm intervals) were selected, including the oocyte nucleus, to quantify glucose transfer. Fluorescence intensity was calculated by measuring total intensity fluorescence (TIF) across three slices, subtracting the fluorescence from denuded oocytes as a measure of passive 2-NBDG transfer.

### 2.8 Statistical analyses

Three independent replicates were conducted, with data presented as percentages or mean ± SD. Statistical analyses were performed using GraphPad Prism 10, with significance set at p < 0.05. Oocyte meiotic competence and parthenogenetic development were evaluated by multiple unpaired t-tests (Holm-Šídák) and one-way ANOVA (Dunnett test). All other data were analyzed using unpaired t-tests.

## 3 Results

### 3.1 SMYD3 activity persists up to 15 h during FEO-IVM and supports meiotic cell cycle progression

**FIGURE 1:**
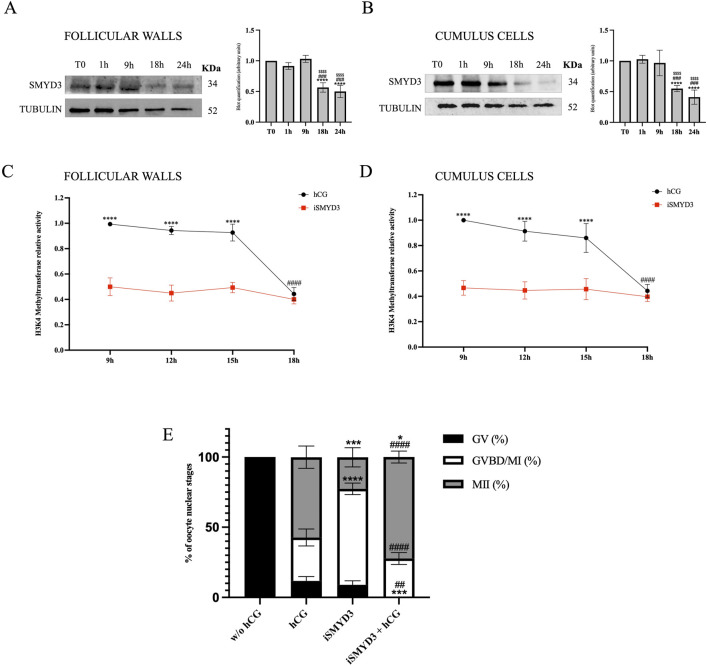
SMYD3 regulation in ovine somatic cells and its functional impact during FEO-IVM. **(A and B)** Immunoblotting analysis of SMYD3 protein levels in follicular walls and cumulus cells collected at 0, 1, 9, and 18 h post-hCG during the FEO-IVM protocol. Tubulin was used as loading control. Bar graphs represent densitometric quantification of SMYD3 normalized to tubulin. Data are expressed as mean ± SD from three independent experiments. * Indicated significance 18 h or 24 h vs. T0 with **** for p < 0.0001; # indicated significance 18 h or 24 vs. 1 h was with ### for p < 0.005; $ indicated significance 18 or 24 h vs. 9 h with $$$$ for p < 0.0001. **(C and D)** Relative H3K4 methyltransferase activity in follicular walls **(A)** and cumulus cells **(B)** collected at 9, 12, 15, and 18 h post-hCG, in the presence or absence of SMYD3 inhibitor (iSMYD3, 200 µM). Data are expressed as mean ± SD from three independent experiments. Statistical significance: * indicated significance vs. between groups (hCG vs iSMYD3) with **** for p < 0.0001; # indicated significance vs. hCG time points with #### for p < 0.0001. **(E)** Nuclear maturation stage of oocytes assessed after 24 h of FEO-IVM in four experimental groups: without hCG (105 EAfs), hCG (25 IU/mL, 101 EAfs), iSMYD3 (200 μM, 98 EAfs), and hCG + iSMYD3 (104 EAfs). Three biological replicates were performed per group. Superscripts indicate statistical significance: iSMYD3 or hCG + iSMYD3 vs. hCG was indicated with **** for p < 0.0001, *** for p < 0.0005 and * for p < 0.05; hCG + iSMYD3 vs. iSMYD3 was indicated with #### for p < 0.0001 and ## for p < 0.05.

The modulation of SMYD3 protein levels in the somatic compartment during FEO-IVM was first assessed by immunoblotting (Figures 1A,B). SMYD3 expression remained stable until 9 h post-hCG stimulation and significantly decreased at 18 h in both follicular walls and cumulus cells (p < 0.0001 vs T0).

Although protein levels dropped at 18 h, the exact timing of SMYD3 enzymatic activity remained unclear between 9 h and 18 h. To better define the active phase of SMYD3 during FEO-IVM, H3K4 methyltransferase activity was measured in protein lysates from cumulus cells and follicular walls collected at various time points between 9 h and 18 h post-hCG, using a colorimetric assay (Figures 1C,D). As a reference for SMYD3 activity, samples treated with iSMYD3 (without hCG) were included. In this condition, enzymatic activity remained consistently low from 9 h onward, thus providing a functional threshold below which SMYD3 can be considered inactive. Based on this comparison, SMYD3 activity was found to persist until 15 h and declined only at 18 h (p < 00,001 vs*.* 15 h).

According to the modulation of SMYD3 presence and activity in the somatic compartments, the role of methyltransferase on oocyte maturation was investigated by using SMYD3 inhibitor EPZ031686 (iSMYD3) during the first 18 h of the EAf FEO-IVM protocol. The dose of the inhibitor (200 µM) was defined by detecting the methylation level of SMYD3 target molecules (Supplementary Figure S1).

The FEO-IVM protocol was ideally to analyze the effect of iSMYD3 on oocyte maturation. Indeed, the meiotic resumption in FEO cultural conditions can be triggered exclusively in the presence of hCG as previously validated (Peserico et al., 2023). More in detail, all oocytes remained quiescent (100% GV) in the absence of any hormonal stimulation (w/o hCG), while most of them (87% MII + GVBD) resumed meiosis after hCG supplementation even if only 57% of them completed the maturation process reaching the MII stage.

Of note, iSMYD3 was able to activate the meiotic cell cycle in the majority of oocytes (91% MII + GVBD), although most of them stopped the meiotic cell cycle at GVBD/MI stage (68%). On the contrary, iSMYD3-treated oocytes progressed towards the MII stage when hCG was supplemented (72.3% vs*.* 23%, *p* < 0.0005; Figure 1E).

### 3.2 SMYD3 expression is modulated according to oocyte maturation outcomes

**FIGURE 2:**
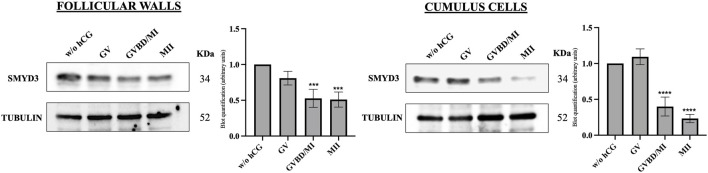
SMYD3 protein expression in the ovine somatic compartments at the end of hCG-induced FEO-IVM of EAfs. SMYD3 expression was analyzed by immunoblotting in cumulus cells and follicular wall isolated from EAf cultured for 24 h without hCG stimulation (w/o hCG) or with hCG stimulation (24 h post-hCG) according to the FEO-IVM protocol. SMYD3 expression data were recorded based on the oocyte nuclear stage (GV, GVBD/MI, MII). A representative immunoblot out of three independent replicates is shown. Data represent the mean of the replicates. Statistical significance: GVBD/MI or MII vs w/o GV was indicated with *** for p < 0.0005 in follicular cells and GVBD/MI or MII vs w/o GV was indicated with **** for p < 0.0001 in cumulus cells.

To better investigate the relationship between SMYD3 expression and oocyte maturation, protein levels were assessed in follicular walls and cumulus cells at the end of FEO-IVM protocol (24 h post-hCG exposure), according to the nuclear stage of the enclosed oocytes (GV, GVBD/MI, and MII). As summarized in Figure 2, meiosis resumption triggered by hCG was achieved with a reduction in SMYD3 levels in both somatic compartments.

In more detail, high SMYD3 levels persisted in EAf that did not support maturation after hCG stimulation (GV oocytes). In contrast, a significant drop in SMYD3 levels was observed in both somatic compartments that successfully resume meiosis after hCG (Figure 2).

### 3.3 iSMYD3 interferes with the parthenogenetic development of matured oocytes

Parthenogenetic activation was performed on FEO-IVM-derived MII oocytes to assess the effect of iSMYD3 on the cytoplasmic quality of matured oocytes.

**FIGURE 3:**
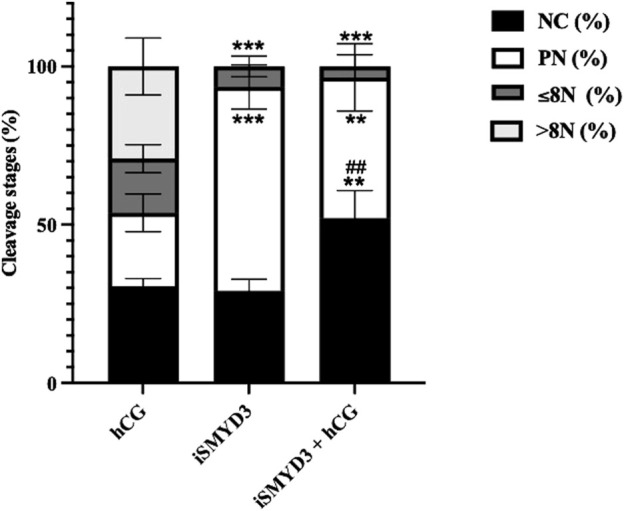
Influence of iSMYD3 on parthenogenetic activation of MII oocytes derived from ovine follicles. Oocyte cleavage was compared 72 h after parthenogenetic activation of MII oocytes obtained from FEO-IVM carried out with: 1) hCG, 2) iSMYD3, 3) hCG + iSMYD3. Parthenogenetic outcomes were identified as no cleaved oocytes (NC), pronuclear (PN) stage, embryos with ≤8 nuclei (≤8N), or >8 nuclei (>8N). Three independent biological replicates were performed. Superscripts indicate statistical significance: iSMYD3 or hCG +iSMYD3 vs. hCG was indicated with **** for p < 0.0001, *** for p < 0.0005 and ** for p < 0.005; hCG + iSMYD3 vs. iSMYD3 was indicated with ## for p < 0.005.

The matured oocytes had a significantly reduced developmental competence when FEO-IVM was performed in the presence of iSMYD3 (Figure 3).

Indeed, the cleavage rate was significantly lower in MII oocytes derived from iSMYD3 group (6% vs*.* hCG; *p* < 0.0005) and no embryo has ever progressed beyond the 8-cell stage. Of note, neither the simultaneous hCG stimulation restored the developmental competence of iSMYD3-treated MII oocytes (Figure 3).

### 3.4 SMYD3 interferes with hCG signaling pathway

**FIGURE 4:**
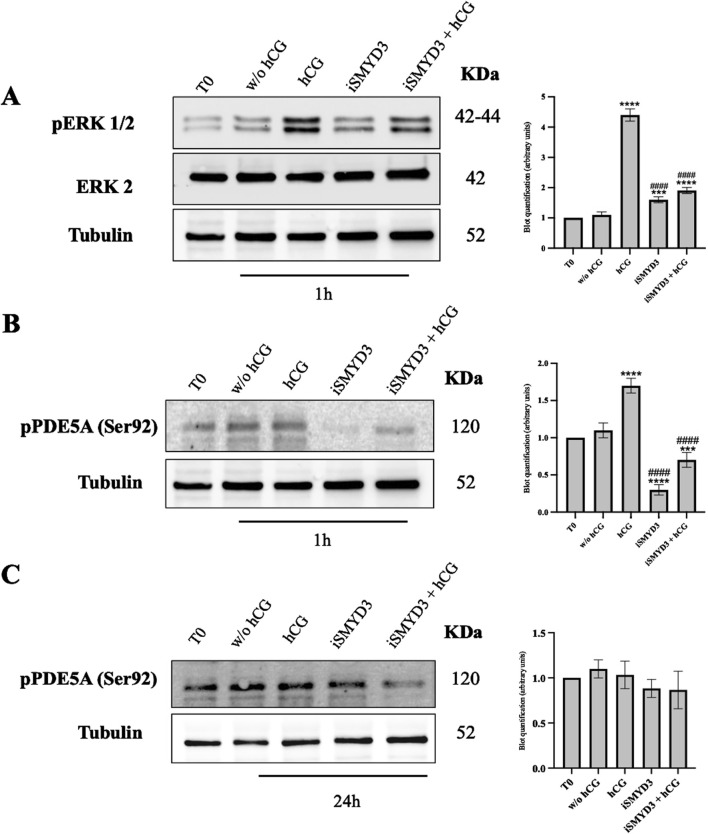
Role of iSMYD3 on MAPK1/3 and PDE5A early activation in ovine cumulus oophorus. **(A)** pERK1/2 expression and **(B and C)** PDE5A phosphorylation at Ser92 were analyzed in COCs collected from freshly isolated EAf (T0) or after 1 or 24 h of FEO. The data are represented as the mean of three biological replicates, with statistical significance indicated: hCG or iSMYD3 or hCG + iSMYD3 vs. w/o hCG was indicated with **** for p < 0.0001, *** for p < 0.005; iSMYD3 or hCG + iSMYD3 vs. hCG was indicated with #### for p < 0.0001.

To determine whether hCG and SMYD3 inhibition promote meiotic resumption through shared or distinct signaling pathways, early intracellular responses in cumulus cells were analyzed. Specifically, the activation of MAPK1/3 (ERK1/2) and phosphorylation of PDE5A at Ser92 were assessed at early time points following FEO-IVM stimulation (Figure 4).

Both pathways were rapidly activated 1 h after hCG stimulation, as evidenced by increased levels of phospho-ERK1/2 (p < 0.0001 vs w/o hCG) and phospho-PDE5A (p < 0.0001 vs w/o hCG). In contrast, SMYD3 inhibition completely prevented ERK1/2 phosphorylation, both in the absence and presence of hCG (p < 0.0001 vs hCG; p > 0.05 iSMYD3 + hCG vs iSMYD3), indicating that hCG-induced ERK activation does not occur when SMYD3 is inhibited (Figures 4A,B).

Similarly, PDE5A phosphorylation was strongly reduced by iSMYD3 treatment (p < 0.0001 vs hCG; p < 0.0001 vs w/o hCG), and was not restored upon hCG addition (p > 0.05 iSMYD3 vs iSMYD3 + hCG). However, this inhibitory effect was not maintained over time: at 24 h, PDE5A phosphorylation levels were comparable across all experimental groups, and similar to those observed at baseline (T0) (Figure 4C).

### 3.5 iSMYD3 induces spontaneous resumption of meiosis by promoting an early activation of CDC25A

**FIGURE 5:**
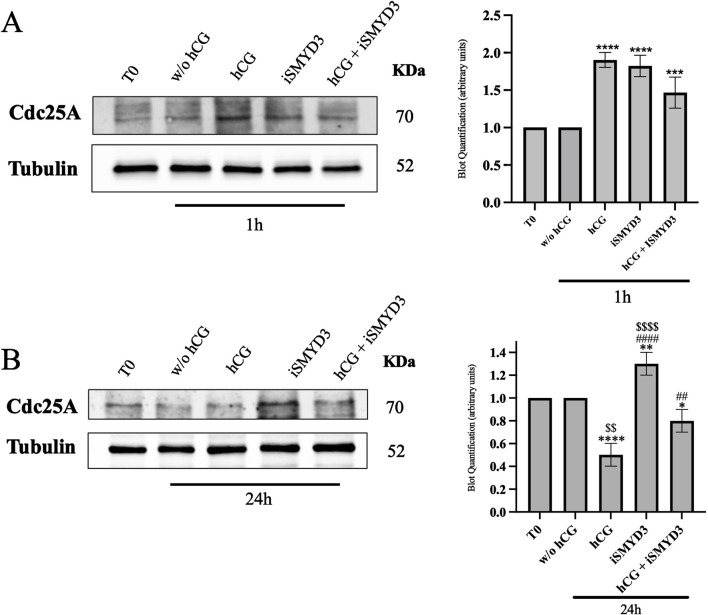
Influence of iSMYD3 on CDC25A expression during ovine oocyte maturation. CDC25A expression was quantified in oocytes collected from freshly isolated EAf (T0) or after 1 h **(A)** and 24 h **(B)** from FEO-IVM carried out: w/o hCG, hCG, iSMYD3, hCG + iSMYD3. The data represent the mean of three biological replicates, with statistical significance indicated: **(A)** hCG or iSMYD3 vs. w/o hCG was indicated with **** for p < 0.0001 and *** for p < 00,005; **(B)** hCG or iSMYD3 or hCG + iSMYD3 vs. w/o hCG was indicated with * for p < 0.005, ** for p < 0.005 and **** for p < 0.0001; iSMYD3 or hCG + iSMYD3 vs. hCG was indicated with ## for p < 0.005 and #### for p < 0.0001; hCG or iSMYD3 vs. hCG + iSMYD3 was indicated with $$ for p < 0.005 and $$$$ for p < 0.0001.

The role of SMYD3 in inducing spontaneous meiosis resumption was assessed by evaluating the expression of a key meiotic cell cycle regulator, such as CDC25A, in oocytes (Figure 5).

As shown in Figure 5A, hCG stimulation induced a rapid upregulation of CDC25A at early time points (1 h) compared to the w/o hCG group (p < 0.0001). A similar increase in CDC25A expression was observed following iSMYD3 treatment (p < 0.0001 vs w/o hCG).

At the end of the FEO-IVM protocol (24 h), CDC25A levels were significantly reduced in oocytes matured with hCG (p < 00,001 vs. w/o hCG; Figure 5B). In contrast, iSMYD3-treated oocytes maintained significantly higher CDC25A levels (p < 0.001 vs hCG). Upon hCG supplementation, CDC25A levels in iSMYD3-treated oocytes were significantly decreased (p < 0.001 vs. iSMYD3 alone), although they remained higher than those observed in oocytes treated with hCG alone (p > 0.005, iSMYD3 + hCG vs. hCG).

### 3.6 iSMYD3 maintained a long-term metabolic coupling between the cumulus and oocyte compartments

**FIGURE 6:**
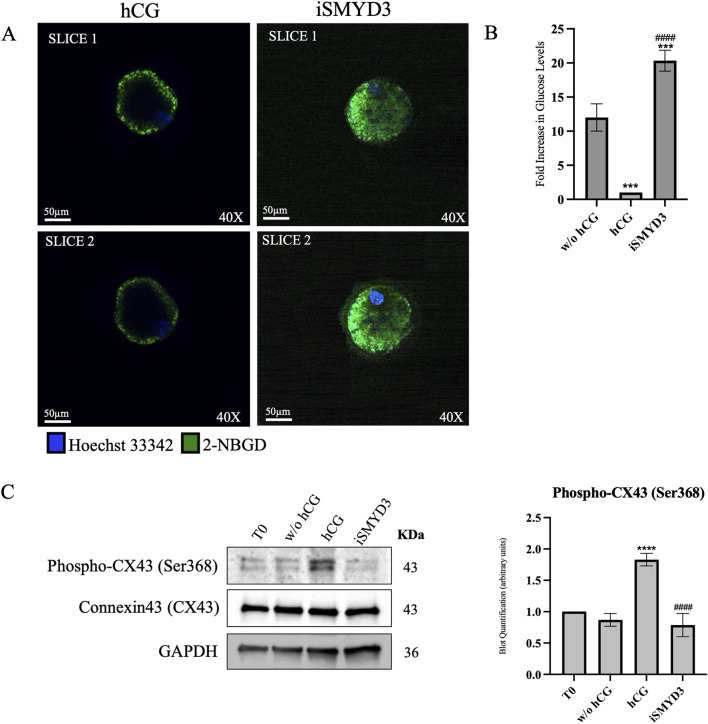
Effect of iSMYD3 on ovine cumulus-oocyte metabolic coupling. **(A)** Confocal images showing 2-NBDG fluorescence (green) as a readout of oocyte glucose uptake in COCs cultured for 18 h under FEO-IVM conditions: positive control (w/o hCG, n = 30), hCG-treated (n = 30), iSMYD3-treated (n = 30). Hoechst 33342 (blue) was used for nuclear staining. After culture, COCs were incubated with 2-NBDG (200 μM, 30 min), denuded, and imaged. Data were normalized to the TIF of the negative control (Ctrl−: denuded oocytes, n = 30). Quantification was performed on two representative slices, spaced 5 µm apart and including the oocyte nucleus. **(B)** Histograms show the TIF of each group subtracted from Ctrl−. Statistical significance: hCG or iSMYD3 vs. w/o hCG was indicated with *** for p < 00,005; iSMYD3 vs. hCG was indicated with #### for 00,001. **(C)** Immunoblot quantification of CX43 and phospho-CX43 (Ser368). Statistical significance: hCG vs. w/o hCG was indicated with **** for p < 0.0001and iSMYD3 vs. hCG was indicated with #### for p < 00,001.

To assess cumulus-oocyte metabolic coupling, the gap junction function was assessed in COCs isolated 18 h after FEO-IVM. To this aim, the transfer of 2-NBDG between cumulus and oocyte compartment was compared after hCG or iSMYD3 treatment (Figures 6A,B).

2-NBDG fluorescence transfer demonstrated that hCG induces a drastic reduction loss in gap junction function in 18 h (10-fold decrease vs. w/o hCG, *p* < 0.0005; see Figures 6A,B). Conversely, a long-term coupling was recorded in iSMYD3-treated COCs where 2-NBDG TIF was even greater than that detected in unstimulated COCs (1.6-fold change increase vs. w/o hCG, *p* < 0.0001 see Figures 6A,B).

Immunoblotting analysis of cumulus cells (Figure 6C) showed that hCG stimulation markedly increased CX43 phosphorylation at Ser368 (p < 0.0001 vs w/o hCG), a modification known to be associated with gap junction closure. These molecular data support the observed impairment of gap junction functionality (Norris et al., 2008). In contrast, phospho-CX43 levels remained unchanged following iSMYD3 treatment, indicating that SMYD3 inhibition does not induce gap junction disruption.

## 4 Discussion

Oocyte maturation is tightly regulated and involves complex interactions between two distinct yet interconnected compartments: the somatic and the germinal. Until the periovulatory stage, meiotic arrest in oocytes is primarily maintained by elevated levels of cyclic AMP (cAMP) induced by the somatic compartment. Indeed, the high levels of cAMP in the oocyte are sustained through extensive communication with surrounding somatic cumulus cells *via* gap junctions (GJs) (Conti and Franciosi, 2018; Xie et al., 2023). These junctions facilitate the transfer of cyclic GMP (cGMP) from somatic cells to the oocyte, where it inhibits phosphodiesterase 3A (PDE3A) activity. This inhibition prevents cAMP degradation, thereby maintaining meiotic arrest by inactivating the maturation-promoting factor (MPF), which is composed of cyclin-dependent kinase 1 (CDK1) and cyclin B (Pei et al., 2023; Pan and Li, 2019).

During each ovarian cycle, the preovulatory surge of LH, or its analogous hCG, initiates meiotic resumption in preovulatory follicles. This hormonal stimulus activates signaling cascades in the somatic compartment—particularly the MAPK pathway and PDE5A activity—gradually reducing cGMP transfer to the oocyte. This reduction, along with the progressive closure of gap junctions, enables PDE3A activation. The resulting decrease in cAMP levels and subsequent activation of MPF triggers GVBD and progression to metaphase II (MII) (Pei et al., 2023).

Given the limited evidence regarding SMYD3’s role in the mechanisms regulating meiosis, numerous findings (Bernard et al., 2021) confirm its involvement in cell cycle regulation across various cellular models, including cancer, where its knockdown leads to reduced proliferation (Zhao et al., 2025; Ren et al., 2011; Asuthkar et al., 2022; Tsai et al., 2016; Sanese et al., 2024; Peserico et al., 2015). Moreover, recent studies suggest that SMYD3 interacts with non-histone proteins, including signaling molecules such as RAF and MEK in the MAPK pathway, further promoting cell proliferation and survival (Bernard et al., 2021; Mazur et al., 2016; Ikram et al., 2023; Fasano et al., 2022). Based on this background, the present study was designed to assess the role of SMYD3 in the intricate processes governing oocyte meiotic arrest and maturation.

The results of this study identify SMYD3 as a novel molecular player in the regulation of meiosis in early ovine follicle structures. Our findings demonstrate, for the first time, that the methyltransferase plays a crucial role in maintaining meiotic arrest within EAFs, as evidenced by the observation that SMYD3 inhibition disrupts the intrafollicular condition of meiotic quiescence, leading to spontaneous oocyte maturation.

Support for SMYD3’s physiological role is further provided by the observation that hCG, which induces oocyte maturation in the FEO-IVM protocol conducted on EAFs (Peserico et al., 2023; Di Berardino et al., 2024), significantly downregulates SMYD3 expression in both the follicular wall and cumulus cell compartments after 18 h post-treatment. This downregulation appears to serve as a permissive signal for meiotic resumption and improved oocyte quality. Notably, a marked reduction in SMYD3 was observed within 18 h after hCG supplementation, but only in follicles containing oocytes that resumed meiosis. In contrast, SMYD3 expression persisted in follicles with oocytes arrested at the GV stage, suggesting a failure in hCG signaling—potentially due to impaired responsiveness of somatic cells or disrupted transmission of maturation cues to the oocyte.

A key mechanism underlying the pro-meiotic effect of SMYD3 inhibition is the significant upregulation of CDC25A, a phosphatase recently shown in cancer cell models to be negatively regulated by SMYD3 through post-translational mechanisms (Jiang et al., 2019). Alongside CDC25B, CDC25A activates MPF, promoting meiotic progression, spindle formation, and the MI to MII transition (Solc et al., 2008; Lincoln et al., 2002; Ferencova et al., 2022). The inverse relationship between iSMYD3 and CDC25A observed in sheep oocytes indicates that SMYD3 functions as a specific suppressor of these phosphatases, which are involved in maintaining meiotic arrest.

However, while increased CDC25A expression following SMYD3 inhibition allows meiosis to resume *via* CDK1 activation, oocytes predominantly arrest at the GVBD/MI stage (Solc et al., 2008; Lincoln et al., 2002; Ferencova et al., 2022). Similar findings were reported in Cdc25b^−/−^ female mice, in which CDK1 activity was restored *via* EGFP-CDC25A (Solc et al., 2008; Lincoln et al., 2002; Ferencova et al., 2022). Furthermore, the precise sequence of CDC25 A/B degradation and reactivation required for the MI–MII transition, as demonstrated in knockout mouse models, appears to necessitate the presence of SMYD3 during the first 18 h of maturation.

While SMYD3 inhibition upregulates CDC25A in the oocyte, promoting meiotic resumption, it is equally important to consider the somatic compartment, where the gonadotropin surge (mimicked by hCG *in vitro*) is initially decoded. The maturation signal originates in the somatic cells and is then relayed to the oocyte. To investigate how SMYD3 influences signaling in the somatic compartment, we examined the effect of its inhibition on two key hCG-induced pathways: MAPK and PDE5A (Egbert et al., 2016; Egbert et al., 2018; Das and Arur, 2022). These pathways are critical mediators of the gonadotropin response in somatic cells, initiating the downstream events that trigger oocyte maturation. Our findings revealed that SMYD3 is also essential for proper MAPK and PDE5A upregulation in response to hCG, suggesting it plays a role in controlling the transmission of the maturation signal from the somatic to the germinal compartment.

These valuable insights into SMYD3’s function was obtained using the FEO-IVM model. Unlike conventional IVM protocols, the FEO model preserves meiotic arrest until active LH/hCG stimulation, allowing for clearer evaluation of SMYD3’s regulatory role. This may explain why earlier studies using standard IVM protocols—such as those by Bai et al. (2016) failed to detect SMYD3’s function, as meiotic resumption occurred spontaneously following cumulus cell removal (Bai et al., 2016).

Beyond meiotic resumption, our results suggest that SMYD3 inhibition, likely by impairing the transmission of hormonal signals to the oocyte, negatively affects developmental competence. Although nuclear maturation was achieved after hCG stimulation, oocytes treated with iSMYD3 exhibited significantly reduced cleavage rates following parthenogenetic activation. This indicates that proper cytoplasmic maturation depends on SMYD3 activity. These findings are consistent with Bai et al. (2016), who observed impaired developmental competence in SMYD-inhibited denuded oocytes without disruption of meiotic progression.

Although the specific mechanism through which SMYD3 regulates the cytoplasmic quality of oocytes that complete maturation is not yet known, it may involve both proper hormonal signal transduction of oocyte maturation pathways—as demonstrated in this study—and final epigenetic remodelling of the maternal genome, both essential to define the oocyte’s developmental potential (Conti and Franciosi, 2018; Bai et al., 2016).

Further underscoring the complexity of SMYD3’s role in mediating the crosstalk between somatic and germinal compartments after hCG stimulation, our study also demonstrated that the methyltransferase regulates intercellular communication between cumulus cells and the oocyte. Following hCG stimulation, connexin 43 (Cx43) undergoes extensive MAPK-mediated phosphorylation, leading to gap junction closure (Norris et al., 2008; Norris et al., 2010; Jaffe and Egbert, 2017). This uncoupling is necessary for meiotic progression. However, under SMYD3 inhibition—despite the resumption of meiosis—gap junction closure was impaired, resulting in prolonged and abnormal communication between compartments. This disruption may further contribute to the reduced developmental competence observed in SMYD3-inhibited oocytes.

## 5 Conclusion

In summary, this study highlights SMYD3 as a modulator of oocyte maturation in sheep, based on protein-level evidence obtained using a 3D FEO-IVM model. SMYD3 inhibition influenced meiotic progression and altered cumulus-oocyte communication, ultimately compromising oocyte developmental competence. While our data provide new functional insights into SMYD3’s involvement in oogenesis, further studies are needed to elucidate the underlying molecular mechanisms, including potential transcriptional or epigenetic regulation.

## Data Availability

The original contributions presented in the study are included in the article/Supplementary Material, further inquiries can be directed to the corresponding author.

## Glossary

SMYD3: SET and MYND domain-containing protein 3
H3K4: Histone 3 lysine 4
GV: Germinal Vesicle
GVBD: Germinal Vesicle Breakdown
MI: Metaphase I
MII: Metaphase II
COC: Cumulus-Oocyte Complex
EAfs: Early Antral Follicles
PCL: Poly(ε-caprolactone)
CDC25A: Cell Division Cycle 25A
MAPK1/3 (ERK1/2): Mitogen-Activated Protein Kinases 1/3 (also known as ERK1/2)
PDE5A: Phosphodiesterase type 5A
CX43: Connexin 43
cAMP: Cyclic Adenosine Monophosphate
cGMP: Cyclic Guanosine Monophosphate
CDK1: Cyclin-Dependent Kinase 1
MPF: Maturation-Promoting Factor
iSMYD3: SMYD3 inhibitor (EPZ031686)
2-NBDG: 2-(N-(7-Nitrobenz-2-oxa-1,3-diazol-4-yl)Amino)-2-Deoxyglucose
FEO: Follicle Enclosed Oocyte
IVM: *In Vitro* Maturation
FEO-IVM: *In Vitro* Maturation of Follicle-Enclosed Oocytes
hCG: Human Chorionic Gonadotropin
LH: Luteinizing Hormone
FSH: Follicle Stimulating Hormone
TIF: Total Intensity Fluorescence
T0: Time zero
PN: Pronuclear stage
≤8N/>8N: Embryos with ≤ or >8 nuclei

## References

[B1] Asuthkar S. Venkataraman S. Avilala J. Shishido K. Vibhakar R. Veo B. (2022). SMYD3 promotes cell cycle progression by inducing cyclin D3 transcription and Stabilizing the cyclin D1 protein in medulloblastoma. Cancers (Basel) 14 (7), 1673. 10.3390/cancers14071673 35406445 PMC8997160

[B2] Bai H. Li Y. Gao H. Dong Y. Han P. Yu H. (2016). Histone methyltransferase SMYD3 regulates the expression of transcriptional factors during bovine oocyte maturation and early embryonic development. Cytotechnology 68 (4), 849–859. 10.1007/s10616-014-9838-5 25563599 PMC4960136

[B3] Barboni B. Russo V. Cecconi S. Curini V. Colosimo A. Garofalo M. L. A. (2011). *In vitro* grown sheep preantral follicles yield oocytes with normal nuclear-epigenetic maturation. PLoS One 6 (11), e27550. 10.1371/journal.pone.0027550 22132111 PMC3221676

[B4] Bernard B. J. Nigam N. Burkitt K. Saloura V. (2021). SMYD3: a regulator of epigenetic and signaling pathways in cancer. Clin. Epigenetics 13 (1), 45. 10.1186/s13148-021-01021-9 33637115 PMC7912509

[B5] Bottino C. Peserico A. Simone C. Caretti G. (2020). SMYD3: an oncogenic driver targeting epigenetic regulation and signaling pathways. Cancers 12 (1), 142. 10.3390/cancers12010142 31935919 PMC7017119

[B6] Clift D. Schuh M. (2013). Restarting life: fertilization and the transition from meiosis to mitosis. Nat. Rev. Mol. Cell Biol. 14 (9), 549–562. 10.1038/nrm3643 23942453 PMC4021448

[B7] Colosimo A. Di Rocco G. Curini V. Russo V. Capacchietti G. Berardinelli P. (2009). Characterization of the methylation status of five imprinted genes in sheep gametes. Anim. Genet. 40 (6), 900–908. 10.1111/j.1365-2052.2009.01939.x 19694650

[B8] Conti M. Franciosi F. (2018). Acquisition of oocyte competence to develop as an embryo: integrated nuclear and cytoplasmic events. Hum. Reprod. Update 24 (3), 245–266. 10.1093/humupd/dmx040 29432538 PMC5907346

[B9] Das D. Arur S. (2022). Regulation of oocyte maturation: role of conserved ERK signaling. Mol. Reprod. Dev. 89 (9), 353–374. 10.1002/mrd.23637 35908193 PMC9492652

[B10] Di Berardino C. Liverani L. Peserico A. Capacchietti G. Russo V. Bernabò N. (2022). When electrospun fiber support matters: *in vitro* ovine long-term folliculogenesis on poly (epsilon caprolactone) (PCL)-Patterned fibers. Cells 11 (12), 1968. 10.3390/cells11121968 35741097 PMC9222101

[B11] Di Berardino C. Peserico A. Camerano Spelta Rapini C. Liverani L. Capacchietti G. Russo V. (2024). Bioengineered 3D ovarian model for long-term multiple development of preantral follicle: bridging the gap for poly(ε-caprolactone) (PCL)-based scaffold reproductive applications. Reprod. Biol. Endocrinol. 22 (1), 95. 10.1186/s12958-024-01266-y 39095895 PMC11295475

[B12] Egbert J. R. Uliasz T. F. Shuhaibar L. C. Geerts A. Wunder F. Kleiman R. J. (2016). Luteinizing hormone causes phosphorylation and activation of the cGMP phosphodiesterase PDE5 in rat ovarian follicles, contributing, together with PDE1 activity, to the resumption of meiosis. Biol. Reprod. 94 (5), 110. 10.1095/biolreprod.115.135897 27009040 PMC4939740

[B13] Egbert J. R. Yee S. P. Jaffe L. A. (2018). Luteinizing hormone signaling phosphorylates and activates the cyclic GMP phosphodiesterase PDE5 in mouse ovarian follicles, contributing an additional component to the hormonally induced decrease in cyclic GMP that reinitiates meiosis. Dev. Biol. 435 (1), 6–14. 10.1016/j.ydbio.2018.01.008 29341896 PMC5818284

[B14] Fasano C. Lepore Signorile M. De Marco K. Forte G. Sanese P. Grossi V. (2022). Identifying novel SMYD3 interactors on the trail of cancer hallmarks. Comput. Struct. Biotechnol. J. 20, 1860–1875. 10.1016/j.csbj.2022.03.037 35495117 PMC9039736

[B15] Ferencova I. Vaskovicova M. Drutovic D. Knoblochova L. Macurek L. Schultz R. M. (2022). CDC25B is required for the metaphase I-metaphase II transition in mouse oocytes. J. Cell Sci. 135 (6), jcs252924. 10.1242/jcs.252924 35237831

[B16] Hamamoto R. Furukawa Y. Morita M. Iimura Y. Silva F. P. Li M. (2004). SMYD3 encodes a histone methyltransferase involved in the proliferation of cancer cells. Nat. Cell Biol. 6 (8), 731–740. 10.1038/ncb1151 15235609

[B17] Ikram S. Rege A. Negesse M. Y. Casanova A. G. Reynoird N. Green E. M. (2023). The SMYD3-MAP3K2 signaling axis promotes tumor aggressiveness and metastasis in prostate cancer. Sci. Adv. 9 (46), eadi5921. 10.1126/sciadv.adi5921 37976356 PMC10656069

[B18] Jaffe L. A. Egbert J. R. (2017). Regulation of mammalian oocyte meiosis by intercellular communication within the ovarian follicle. Annu. Rev. Physiol. 79, 237–260. 10.1146/annurev-physiol-022516-034102 27860834 PMC5305431

[B19] Jiang Y. Lyu T. Che X. Jia N. Li Q. Feng W. (2019). Overexpression of SMYD3 in ovarian cancer is associated with ovarian cancer proliferation and apoptosis via methylating H3K4 and H4K20. J. Cancer 10 (17), 4072–4084. 10.7150/jca.29861 31417652 PMC6692630

[B20] Lincoln A. J. Wickramasinghe D. Stein P. Schultz R. M. Palko M. E. De Miguel M. P. (2002). Cdc25b phosphatase is required for resumption of meiosis during oocyte maturation. Nat. Genet. 30 (4), 446–449. 10.1038/ng856 11912493

[B21] Liu C. Fang X. Ge Z. Jalink M. Kyo S. Björkholm M. (2007). The telomerase reverse transcriptase (hTERT) gene is a direct target of the histone methyltransferase SMYD3. Cancer Res. 67 (6), 2626–2631. 10.1158/0008-5472.CAN-06-4126 17363582

[B22] Mazur P. K. Gozani O. Sage J. Reynoird N. (2016). Novel insights into the oncogenic function of the SMYD3 lysine methyltransferase. Transl. Cancer Res. 5 (3), 330–333. 10.21037/tcr.2016.06.26 30713830 PMC6358167

[B23] Norris R. P. Freudzon M. Mehlmann L. M. Cowan A. E. Simon A. M. Paul D. L. (2008). Luteinizing hormone causes MAP kinase-dependent phosphorylation and closure of connexin 43 gap junctions in mouse ovarian follicles: one of two paths to meiotic resumption. Development 135 (19), 3229–3238. 10.1242/dev.025494 18776144 PMC2572224

[B24] Norris R. P. Freudzon M. Nikolaev V. O. Jaffe L. A. (2010). Epidermal growth factor receptor kinase activity is required for gap junction closure and for part of the decrease in ovarian follicle cGMP in response to LH. Reproduction 140 (5), 655–662. 10.1530/REP-10-0288 20826538 PMC3119707

[B25] Oliveri R. S. Kalisz M. Schjerling C. K. Andersen C. Y. Borup R. Byskov A. G. (2007). Evaluation in mammalian oocytes of gene transcripts linked to epigenetic reprogramming. Reproduction 134 (4), 549–558. 10.1530/REP-06-0315 17890290

[B26] Pan B. Li J. (2019). The art of oocyte meiotic arrest regulation. Reprod. Biol. Endocrinol. 17 (1), 8. 10.1186/s12958-018-0445-8 30611263 PMC6320606

[B27] Pei Z. Deng K. Xu C. Zhang S. (2023). The molecular regulatory mechanisms of meiotic arrest and resumption in Oocyte development and maturation. Reprod. Biol. Endocrinol. 21 (1), 90. 10.1186/s12958-023-01143-0 37784186 PMC10544615

[B28] Peserico A. Di Berardino C. Capacchietti G. Camerano Spelta Rapini C. Liverani L. Boccaccini A. R. (2023). IVM advances for early antral follicle-enclosed oocytes coupling reproductive tissue engineering to inductive influences of human chorionic gonadotropin and ovarian surface epithelium coculture. Int. J. Mol. Sci. 24 (7), 6626. 10.3390/ijms24076626 37047595 PMC10095509

[B29] Peserico A. Germani A. Sanese P. Barbosa A. J. Di Virgilio V. Fittipaldi R. (2015). A SMYD3 small-molecule inhibitor impairing cancer cell growth. J. Cell Physiol. 230 (10), 2447–2460. 10.1002/jcp.24975 25728514 PMC4988495

[B30] Phillips T. C. Wildt D. E. Comizzoli P. (2016). Incidence of methylated histones H3K4 and H3K79 in cat germinal vesicles is regulated by specific nuclear factors at the acquisition of developmental competence during the folliculogenesis. J. Assist. Reprod. Genet. 33 (6), 783–794. 10.1007/s10815-016-0706-4 27059775 PMC4889483

[B31] Ren T. nian Wang J. song He Y. mian Xu C. liang Wang S. zhen Xi T. (2011). Effects of SMYD3 over-expression on cell cycle acceleration and cell proliferation in MDA-MB-231 human breast cancer cells. Med. Oncol. 28 (S1), 91–98. 10.1007/s12032-010-9718-6 20957523

[B32] Rubio-Tomás T. (2021). Novel insights into SMYD2 and SMYD3 inhibitors: from potential anti-tumoural therapy to a variety of new applications. Mol. Biol. Rep. 48 (11), 7499–7508. 10.1007/s11033-021-06701-6 34510321

[B33] Russo V. Berardinelli P. Martelli A. Di Giacinto O. Nardinocchi D. Fantasia D. (2006). Expression of telomerase reverse transcriptase subunit (TERT) and telomere sizing in pig ovarian follicles. J. Histochem Cytochem 54 (4), 443–455. 10.1369/jhc.4A6603.2006 16400001

[B34] Russo V. Martelli A. Berardinelli P. Di Giacinto O. Bernabò N. Fantasia D. (2007). Modifications in chromatin morphology and organization during sheep oogenesis. Microsc. Res. Tech. 70 (8), 733–744. 10.1002/jemt.20462 17394198

[B35] Sanese P. Fasano C. Buscemi G. Bottino C. Corbetta S. Fabini E. (2020). Targeting SMYD3 to sensitize homologous recombination-proficient tumors to PARP-mediated synthetic lethality. iScience 23 (10), 101604. 10.1016/j.isci.2020.101604 33205017 PMC7648160

[B36] Sanese P. Fasano C. Lepore Signorile M. De Marco K. Forte G. Disciglio V. (2024). Methyltransferases in cancer drug resistance: unlocking the potential of targeting SMYD3 to sensitize cancer cells. Biochim. Biophys. Acta Rev. Cancer 1879 (6), 189203. 10.1016/j.bbcan.2024.189203 39461625

[B37] Solc P. Saskova A. Baran V. Kubelka M. Schultz R. M. Motlik J. (2008). CDC25A phosphatase controls meiosis I progression in mouse oocytes. Dev. Biol. 317 (1), 260–269. 10.1016/j.ydbio.2008.02.028 18367163 PMC2430978

[B38] Suzuki S. Nozawa Y. Tsukamoto S. Kaneko T. Imai H. Minami N. (2015). Histone methyltransferase Smyd3 regulates early embryonic lineage commitment in mice. Reproduction 150 (1), 21–30. 10.1530/REP-15-0019 25918436

[B39] Tsai C. H. Chen Y. J. Yu C. J. Tzeng S. R. Wu I. C. Kuo W. H. (2016). SMYD3-Mediated H2A.Z.1 methylation promotes cell cycle and cancer proliferation. Cancer Res. 76 (20), 6043–6053. 10.1158/0008-5472.CAN-16-0500 27569210

[B40] Xie J. Xu X. Liu S. (2023). Intercellular communication in the cumulus-oocyte complex during folliculogenesis: a review. Front. Cell Dev. Biol. 11, 1087612. 10.3389/fcell.2023.1087612 36743407 PMC9893509

[B41] Zhao W. W. Gao Y. Zhu Y. T. Zhong F. L. Luo X. G. (2025). SMYD3 plays a pivotal role in mediating the epithelial-mesenchymal transition process in breast cancer. Biochem. Biophysical Res. Commun. 749, 151363. 10.1016/j.bbrc.2025.151363 39864383

