# Supplementary material for: Investigating SMYD3 role during oocyte maturation in a 3D follicle-enclosed oocyte in vitro model in sheep
Source: Front Cell Dev Biol. 2025 Jun 25;13:1625914. doi: 10.3389/fcell.2025.1625914 (PMC12238014; doi:10.3389/fcell.2025.1625914)
Supplement: Supplementary file 1 [file DataSheet1.docx]

Supplementary Material

**Supplementary Table 1: List of antibodies used for Western Blot analysis.** The table provides details on the antibodies used in the experiments, including the target antigen, supplier, product code, host species, and the dilutions applied. Primary antibodies were used to detect specific proteins, while secondary antibodies, conjugated with horseradish peroxidase (HRP), were used for chemiluminescent detection. Dilution factors were optimized based on the experimental conditions and antibody specifications

| **Antibody** | **Supplier** | **Product code** | **Host species** | **Dilution** | **Antibody type** |
| --- | --- | --- | --- | --- | --- |
| Anti-αTubulin | Cell Signaling Technology, Danvers, MA, USA | #3873 | Mouse | 1:1000 | Primary |
| Anti-GAPDH | Sigma Chemical Co., St. Louis, MO, USA | G9545 | Rabbit | 1:2000 | Primary |
| Anti-Histone H3 | Abcam Plc, Cambridge, United Kingdom | ab1791 | Rabbit | 1:1000 | Primary |
| Anti-Di-Methyl-Histone H3 (Lys4) | Cell Signaling Technology, Danvers, MA, USA | #9725 | Rabbit | 1:1000 | Primary |
| Anti-SMYD3 | Cell Signaling Technology, Danvers, MA, USA | #12859 | Rabbit | 1:1000 | Primary |
| Anti-pERK | Cell Signaling Technology, Danvers, MA, USA | #9101 | Rabbit | 1:1000 | Primary |
| Anti-ERK | Santa Cruz Biotechnology, Dallas, TX, USA | sc-154 | Rabbit | 1:1000 | Primary |
| Anti-PDE5A (phospho Ser 92) | GeneTex, Irvine, CA, USA | GTX36930 | Rabbit | 1:1000 | Primary |
| Anti-cdc25A | Cell Signaling Technology, Danvers, MA, USA | #3652 | Rabbit | 1:1000 | Primary |
| Anti-Phospho-Connexin 43 (Ser368) | Invitrogen - Thermo Fisher Scientific, US | 483000 | Mouse | 1:1000 | Primary |
| Anti-Connexin 43 | Santa Cruz Biotechnology, Dallas, TX, USA | sc-271837 | Rabbit | 1:1000 | Primary |
| Anti-mouse IgG HRP | Santa Cruz Biotechnology, Dallas, TX, USA | sc-516102 | Rabbit | 1:4000 | Secondary HRP-conjugated |
| Anti-rabbit IgG HRP | Cytiva, Marlborough, MA, USA | NA934 | Goat | 1:10000 | Secondary HRP-conjugated |

## Supplementary Figure


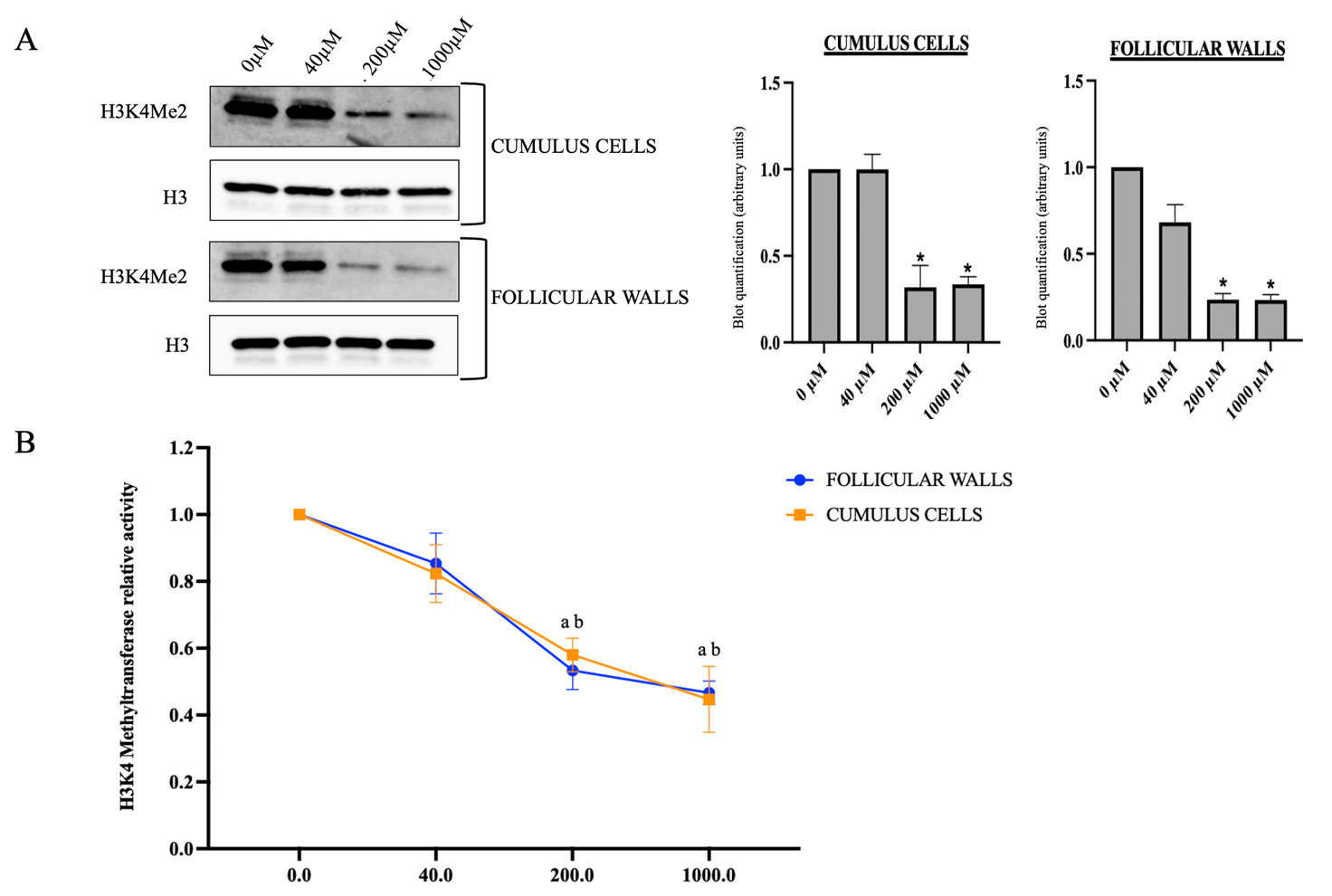


**Supplementary Figure 1. Dose–response effect of EPZ031686 on H3K4me2 levels.**

(A) EA follicles were treated for 18h with 0, 40, 200, or 1000 µM EPZ031686. H3K4me2 levels were analyzed in cumulus cells and follicular walls. H3 was used for normalization. Data represent the mean ± SD of three independent experiments. *p < 0.0001 vs. 0 µM.*p < 0.0001 vs. 0 µM. (B) Relative H3K4 methyltransferase activity measured in follicular walls and cumulus cells after 24 h of FEO-IVM with increasing concentrations of iSMYD3 (0, 40, 200, 1000 µM). Data are expressed as mean ± SD from three independent experiments. Letters (a for Follicular walls; b for Cumulus cells) denote statistically significant differences across concentrations (p < 0.0001, intragroup comparison).
